# A national survey of Rett syndrome: behavioural characteristics

**DOI:** 10.1186/s11689-015-9104-y

**Published:** 2015-03-04

**Authors:** Rina Cianfaglione, Angus Clarke, Michael Kerr, Richard P Hastings, Chris Oliver, Jo Moss, Mary Heald, David Felce

**Affiliations:** Welsh Centre for Learning Disabilities, Institute of Psychological Medicine and Clinical Neurosciences, Cardiff University, 2nd floor Hadyn Ellis Building, Maindy Road, Cardiff, CF24 4HQ, UK; Institute of Cancer & Genetics, Cardiff University, Institute of Medical Genetics Building, Heath Park, Cardiff, CF14 4XN UK; Centre for Educational Development Appraisal and Research, University of Warwick, Coventry, CV4 7AL UK; Cerebra Centre for Neurodevelopmental Disorders, School of Psychology, University of Birmingham, Birmingham, B15 2TT UK

**Keywords:** Intellectual disabilities, Rett syndrome, Behavioural characteristics, Great Britain

## Abstract

**Background:**

The aim was to gain a UK national sample of people with Rett syndrome (RTT) across the age range and compare their characteristics using a variety of relevant behavioural measures with a well-chosen contrast group.

**Methods:**

The achieved sample was 91 girls and women, aged from 4 to 47 years, of whom 71 were known to be *MECP2* positive. The contrast group (*n* = 66), matched for age, gender, language and self-help skills, comprised individuals with six other syndromes associated with intellectual disability. Parental questionnaire measures of RTT specific characteristics, impulsivity, overactivity, mood, interest and pleasure, repetitive behaviour and self-injury were administered.

**Results:**

Hand stereotypies, breathing irregularities, night-time unrest and anxiety or inappropriate fear were commonly reported among the RTT sample. Problems of low mood were also reported as common. However, mood and interest and pleasure were no lower than found in the contrast group. In addition, self-injury was lower than in the contrast group and was associated with factors found to predict self-injury in other groups of people with severe intellectual disabilities.

**Conclusions:**

There is variability in the manifestation of problem behaviours potentially associated with the syndrome across individuals, with some more severely affected in most areas than others. Some of this variability appears to be underpinned by genetic mutation.

## Background

Rett syndrome (RTT) is a genetic disorder that causes severe cognitive and physical impairments. In its classic form, it appears to affect almost exclusively females, with an incidence of up to one in every 10,000 live female births. Its cause is most often a mutation in the methyl-CpG binding protein-2 (*MECP2*) gene, located on the X chromosome at *Xq28* [1]. However, although a *MECP2* mutation is found in most cases of the classic form, RTT remains a clinical rather than a molecular diagnosis. *MECP2* mutations have not been found in all cases of RTT, and mutation has been found in individuals who do not meet the clinical diagnostic criteria for classic or variant RTT [2].

Neul *et al*. [3] described revised diagnostic criteria. Classic RTT requires apparently normal psychomotor development in the first 6 months of life followed by a period of regression, which is not due to brain injury secondary to trauma, neurometabolic disease or severe infection, and involves partial or complete loss of acquired purposeful hand skills and language, gait abnormalities and the development of stereotypic hand movements, followed by stabilization or even some degree of recovery. An important aspect of the regression is a period of social withdrawal or impaired communication. Atypical RTT requires a similar period of regression and subsequent stabilization/recovery, at least two of the above four behavioural manifestations and the presence of at least five (out of 11) supportive criteria. Other variant forms have also been described [3].

The presence of certain behavioural features in the main or supportive diagnostic criteria suggests that RTT syndrome has a definable behavioural phenotype. The most evident characteristics of a RTT behavioural phenotype are the development of repetitive hand stereotypies, such as wringing, clapping or washing hands, together with a loss of functional hand use [4]. Additional potential aspects are social withdrawal, autistic features, bruxism, breathing abnormalities (deep breathing, apnea, hyperventilation, valsava manouvre) and sleep disturbances [5,6]. Table 1 lists behaviours mentioned as occurring either frequently or fairly frequently in five surveys of RTT. Hand stereotypies appear to be pervasive when assessed. Teeth grinding, sleeping difficulties and night-time laughing, screaming, anxiety or inappropriate fear, problems in mood regulation, breathing abnormalities and self-injury may also be expected in the majority or substantial minority. Mount *et al*. [7] reported that repetitive hand movements, breathing problems, signs of fear/anxiety, screaming, crying and laughing at night-time, repetitive mouth/tongue movements and facial grimacing were more frequently reported in a RTT group than a contrast group comprising individuals with severe or profound intellectual disabilities.

**Table 1:** **Behavioural commonalities between surveys of RTT syndrome**

**Behavioural characteristic**	**Percentage of sample with characteristic**					
	**Coleman et al.** **[** 8 **]**	**Samson et al. [** 9 **]**	**Mount et al.** **[** 6 **]**	**Cass et al. [** 10 **]**	**Halbach et al.** **[** 11 **]**	**This sample**
	**(** ***N*** **= 63)**	**(** ***N*** **= 107)**	**(** ***N*** **= 38)**	**(** ***N*** **= 87)**	**(** ***N*** **= 53)**	**(** ***N*** **= 91)**
Hand stereotypies	100	-	100	97	-	99
Teeth grinding	95	-	37	-	-	58
Screaming	84	48 (night)	-	-	39 (night)	44
Night unrest/laughing	83	84	21	-	77	64
Anxiety/inappropriate fear	75	75	-	-	68	73
Low mood/mood changes	-	70	-	-	66	77
Hyperventilation	63	32	84	60	39	63
Breath hold	57	-	37	41	73	77
Self-injury	49	48	-	73	-	28

However, the existing literature has some limitations. First, RTT is rare and survey sizes are necessarily small. There is a need for further research to increase the evidence base. Second, there is a greater representation of children than adults in existing surveys. There is a need for further research on the behavioural characteristics of adults and on developmental trajectory into adulthood. Third, studies lack well-matched contrast groups in comparison to which a distinctive behavioural phenotype might be established. Fourth, certain behaviours, such as impulsivity, overactivity and withdrawal have received relatively less research attention. Impulsivity and overactivity are important to explore in those with severe or profound intellectual disability for their association with self-injury and aggression [12]. Depression in RTT has never been researched. Its assessment in this group is a challenge due to characteristic profound intellectual disability and the associated inability to self-report feelings and emotions. One approach is to assess the presence of abnormally low mood and lack of interest [13,14].

The purpose here was, therefore, to gain a UK national sample of people with Rett syndrome across the age range, use a variety of relevant behavioural measures and compare their characteristics with a contrast group, controlled for gender, age, language and functional ability.

## Methods

### Survey sample

Before commencing the study, ethical approval was granted by the Research Ethics Committee for Wales (Application number: 09/MRE09/50).

The survey methodology is described in greater detail in Cianfaglione *et al*. [15]. In brief, families were recruited through the British Isle Rett Syndrome Survey (BIRSS), an on-going database now maintained by AC at Cardiff University. Families (308) with a daughter or son with RTT were approached and 126 (40.9%) returned a consent form. Questionnaire packs were then distributed and families were contacted first by telephone and then by letter if they had not returned the questionnaires within 2 months from receiving them. Ninety-three families returned completed questionnaires (30.2% of the original 308, 73.8% of those who consented to take part). Ninety-two participants with RTT were female and one was male. The male participant was excluded from the final sample. One participant passed away during the study and was not included in the analysis.

### Sample characteristics

The achieved sample comprised 91 girls and women with a diagnosis of RTT, of whom 80 (87.9%) lived at home and 11 (12.1%) lived in out of family placements. The sample was skewed towards people living in the family home as another research aim was to investigate the well-being of parents caring for a child with RTT (although this survey sought to include only individuals living with their parents, the information on the BIRSS database was not entirely up-to-date and a minority no longer did so). Ages ranged from 4 to 47 years with a mean of 20.5 years: 43 participants were children and 48 adults. Sixty nine had classic RTT (75.8%), 19 atypical RTT (20.9%) and three a *MECP2*-related disorder (4.3%). Seventy one were known to be *MECP2* positive (78.0%): 52 in the classic group and 16 in the atypical group in addition to the three with *MECP2*-related disorder. Diagnosis of RTT was made by a pediatrician in 42.9% of cases, a clinical geneticist in 26.4%, by both a pediatrician and clinical geneticist in 3.3% and by another professional in 25.3% (this information was missing for the remaining 2.2%). Median age of diagnosis was 3.0 years (range, 1 to 39 years). Diagnosis occurred most commonly between 2 and 4 years of age.

Regression was reported in 87 (95.6%). In one case (1.1%), the mother was not sure if the child had had a regression and, in 3 others (3.3%), all with *MECP2*-related disorder, they reported that the child did not have a regression. Mean age of regression was 18.9 months (range, 6 to 84 months; SD 11.75): 15 (16.5%) had a regression before 12 months, 49 (53.8%) between 12 and 18 months, 18 (19.0%) between 19 and 36 months and 5 (5.5%) after 36 months (including, one participant who had a late regression at 7 years).

### Contrast group

The Cerebra Centre, University of Birmingham, has gathered behavioural data over many years on individuals with intellectual disability associated with a variety of genetic syndromes other than RTT [16,17]. Access to these data enabled a contrast group (*n* = 66) to be selected that closely matched the RTT sample. Groups were matched on (a) gender, (b) chronological age, (c) mobility, (d) the self-help skills of feeding, washing and dressing and (e) use of words. The latter was a key matching criterion and only individuals with no verbal ability were included. Hence, the three individuals in the RTT sample with preserved verbal ability were excluded from the comparison made to the contrast group, reducing the RTT group to 88 for this aspect of the analysis. In other respects, all participants were female. On average, the RTT sample were 20.3 years old (SD 10.2; range, 4 to 47 years) and the contrast group 15.0 years old (SD 10.0; range, 4 to 45 years). Fifty percent of the RTT group were mobile independently compared to 53.0% of the contrast group. Just a third of the RTT sample (37.5%) could feed themselves with help compared to 68.2% of the contrast group. No-one in either the RTT sample or contrast group could feed themselves independently or wash or dress themselves either independently or with help.

The contrast group comprised individuals with Cornelia de Lange syndrome (*N* = 26, 39.4%), Angelman syndrome (*N* = 25, 37.9%), Cri du Chat syndrome (*N* = 5, 7.6%), 1p36 deletion syndrome (N = 7, 10.6%), Smith Magenis syndrome (*N* = 2, 3.0%) and Prader Willi syndrome (*N* = 1, 1.5%).

### Measurement

Families were asked to complete two questionnaire packs. One questionnaire pack related to the person with RTT, covering their early development, current skills, health and behavioural characteristics. The second questionnaire pack related to various aspects of family experience. It is some of the first set of measures that are of concern here. Most of the chosen measures had been developed and used by the Cerebra Centre in their research. However, the first two measures listed below were RTT specific and were, therefore, not available for the contrast group.

#### Simplified severity score

In the simplified severity score [18], information was requested about six features of RTT (sitting, walking, hand use, speech, epilepsy and spine deformation). Each domain is scored from 0 to 3, where 0 indicates a normal situation, 1 indicates impaired ability to sit and walk, reduced hand use, some words, epilepsy is controlled with medication and scoliosis is mild; 2 indicates that the abilities to sit, walk, use hands and speak are lost, epilepsy is uncontrolled and scoliosis is severe; 3 indicates that the individual never acquired the abilities to sit, walk, use hands and speak, status epilepticus occurs and scoliosis has been operated upon. The severity score evaluates the overall severity of the syndrome and indicates domains that are considered to influence evolution and severity in the long term. However, it is not sensitive to progression of the syndrome over time. The maximum score is 18. Cases with a score less than 9 are considered mild or less severe.

#### Rett syndrome behavioural questionnaire

The Rett Syndrome Behavioural Questionnaire (RSBQ) [19] is a 45-item checklist developed to assess behavioural and emotional characteristics of RTT. Items are rated 0 to 2, where 0 indicates that the behaviour is not true, 1 sometimes true and 2 often true. The scale is divided into eight subscales: General Mood, Breathing Abnormalities, Hand Behaviours, Repetitive Face Movements, Body Rocking and Expressionless Face, Night-time Behaviour, Fear/Anxiety and Walking/Standing. High internal consistency has been reported for the total score (>0.90) and eight subscales (0.60 to 0.79), with good inter-rater and test-retest reliability scores (total score, >0.80; subscales, 0.60 to 0.79) [19].

#### Activity questionnaire

The Activity Questionnaire (AQ) [20] is an informant-based questionnaire that measures the frequency of impulsivity and overactivity behaviour in children and adults with intellectual disabilities, with or without verbal communication and mobility. It contains 18 questions (for example, Does your child wriggle or squirm about when seated or laying down? Does your child find it difficult holding still?) rated on a five-point Likert scale, where 0 indicates never or almost never, 1 some of the time, 2 half of the time, 3 a lot of the time and 4 always or almost all the time. Behavioural features are clearly described and the respondent is asked to rate the frequency of each behaviour in the last 4 weeks. The scale is divided into three subscales: Overactivity, Impulsivity and Impulsive Speech.

Immobile and non-verbal individuals are scored differently from those who can walk and/or speak. Scores on the Impulsivity subscale for non-mobile individuals are pro-rated to compare with those for mobile individuals. Good internal consistency, item level inter-rater reliability score ranges of 0.31 to 0.75 (mean 0.56) and test re-test reliability score ranges of 0.60 to 0.90 (mean 0.75) have been reported across the subscales [20].

#### Mood, interest and pleasure questionnaire short-form

The Mood, Interest and Pleasure Questionnaire Short-Form (MIPQ-S) [21] assesses mood, interest and pleasure levels in individuals with severe and profound intellectual disability. It contains 12 items scored using a five-point Likert scale based on the respondents’ observation of the participant in the last 2 weeks. High scores indicate positive mood and high interest and pleasure. There are two subscales: Mood and Interest and Pleasure. Scores up to and including 15 and 6 (≤18 years) and 13 and 6 (>18 years) have been identified as being abnormally low and scores equal to or above 24 and 23 (≤18 years) and 24 and 21 (<18 years) as being abnormally high for the two subscales, respectively [22]. Inter-rater and test-retest reliability scores have been reported as good (0.85 and 0.97 respectively) as has internal consistency (Cronbach’s alpha coefficient Total = 0.88, Mood = 0.79, Interest and Pleasure = 0.87) [21].

#### Repetitive behaviour questionnaire

The Repetitive Behaviour Questionnaire (RBQ) [17] is a 19-item informant-based scale used to assess repetitive behaviour in individuals with intellectual disability. It has five subscales: Stereotyped Behaviour, Compulsive Behaviour, Restricted Preferences, Repetitive Use of Language, and Insistence on Sameness. However, the Repetitive Use of Language and Restricted Preferences subscales cannot be scored for individuals with no language as items require the person to be verbal. The frequency of behaviour on each item is scored on a five-point Likert scale (0 to 4). Two scoring systems can be applied for verbal (total score range, 0 to 76) and non-verbal individuals (total score range, 0 to 60). Items that are dependent on the person being verbal can be excluded when comparing verbal and non-verbal individuals. Clinical cut-offs for each subscale are reached if the individual scores three or more on at least one item (that is, a behaviour occurs ‘once a day’ or ‘more than once a day’). Inter-rater reliability scores ranging from 0.46 to 0.80 at item level and test-retest reliability scores ranging from 0.61 to 0.93 at item level have been reported [13]. The following internal consistency coefficients have been reported: full-scale level > .80, the stereotyped behavior and compulsive behavior subscales both > .70, restricted preferences, repetitive speech and insistence on sameness subscales .50, .54 and .65, respectively [17].

#### Challenging behaviour questionnaire

The Challenging Behaviour Questionnaire (CBQ) [23] is an informant-based scale that assesses the presence and frequency of self-injury and aggressive behaviour. Respondents are asked to rate the presence of self-injury and aggression in the last month and to specify the topography of the self-injurious behaviour (hitting self, bites self, slap, bangs head, pulls hair or skin, rubs or scratches self, inserts finger or objects in self). Psychometric properties of the scale are considered to be good with inter-rater reliability coefficients ranging from 0.61 to 0.89 [23].

### Data analysis

For a few participants, some questionnaire items were missing even after attempting to complete them by contacting the respondents by telephone or using relevant information provided in response to another question. Guidelines from questionnaire manuals were employed for pro-rating missing data. Where the missing items were part of a scale or subscale, the mean for the scale/subscale was substituted, providing that 75% of items were scored for the MIPQ and AQ, 65% of items in each subscale were rated for the RBQ and 90% of items were rated for the RSBQ. Having done this, one case was excluded from the analysis of both the RSBQ and AQ and two from the analysis of the MIPQ due to missing data.

Total and subscale mean scores for the RSBQ, AQ, MIPQ and RBQ were calculated. Two sets of analyses were then pursued: (a) testing to establish differences between the RTT sample and the contrast group, and (b) exploration of variation within the RTT group in relation to clinical severity (more vs. less severe) and mutation groups. Although data on the *MECP2* mutation were available, six broad categories were created to avoid subgroups being too small: Missense (*n* = 23, 25.3%), Early Truncating (*n* = 26, 28.6%), Late Truncating (*n* = 7, 7.7%), C-Terminal (*n* = 13, 14.3%), Large Deletion (*n* = 2, 2.2%) and No Known Mutation (*n* = 20, 22.0%). Disregarding the last category, the remaining five categories were then combined into two broader mutation groups: (a) Early Truncating and Large Deletion, and (b) Missense, Late Truncating and C-Terminal, in line with the findings of Neul *et al*. [24]. Cross tabulation with associated chi-squared tests, non-parametric Mann–Whitney *U* tests or Kruskal-Wallis analysis of variance with *post hoc* Mann–Whitney *U* tests together with non-parametric (Spearman) correlations were used to explore differences between groups and relationships between variables.

## Results

### Behavioural characteristics of the RTT sample

The percentage occurrence of potentially characteristic behaviour in this RTT sample is also given in Table 1, alongside findings from the five previous surveys mentioned in the introduction. Hand stereotypies were almost universal (99%). Teeth grinding (58%), sleeping difficulties and night-time laughing (64%), anxiety or inappropriate fear (73%), low mood/changeable mood (77%), breath holding (63%) and hyperventilation (77%) were reported among the majority.

Table 2 sets out RSBQ total and subscale scores. Means were generally near half of the maximum scores possible and ranges were broad. As Table 3 shows, there was a high degree of positive intercorrelation between all RSBQ subscales other than the Walking/Standing domain. RSBQ scores were not significantly associated with the simplified severity score, except in the case of the Walking/Standing domain where scores were higher among those with less severe clinical characteristics (*U* = 319.50, *z* = −5.676, *P* < .001). RSBQ total and subscale scores were also not significantly associated with mutation categories as initially set out. However, there were significant differences in two of the three stereotypy domains between the two broader mutation groups. Scores for Hand Behaviours and Repetitive Face Movements were greater among those with Early Truncating or Large Deletion mutations compared to those with Missense, Late Truncating or C-Terminal mutations (*U* = 415.5, *z* = −2.220, *P* < .05 and *U* = 415.0, *z* = −2.340, *P* < .05). Mean score for Hand Behaviours for the former was 9.1 (SD 1.86) compared to 7.8 (SD 2.39) and for Repetitive Face Movements was 3.2 (SD 1.79) compared to 2.3 (SD 1.82).

**Table 2:** **RSBQ total and subscale mean scores, SDs, ranges and maximum scores**

**RSBQ**	**Mean (SD)**	**Range**
Total (max = 90)	42.31 (14.85)	12 to 78
General mood (max = 16)	6.48 (3.98)	0 to 16
Breathing problems (max = 10)	5.05 (3.06)	0 to 10
Hand behaviours (max = 12)	8.44 (2.36)	1 to 12
Repetitive face movements (max = 8)	2.91 (1.94)	0 to 8
Body rocking and expressionless face (max = 12)	5.41 (2.23)	1 to 12
Night-time behaviours (max = 6)	1.74 (1.59)	0 to 6
Fear/anxiety (max = 8)	4.40 (2.24)	0 to 8
Walking/standing (max = 8)	1.31 (1.46)	0 to 4

RSBQ, rett syndrome behavioural questionnaire.

**Table 3:** **Non-parametric correlation matrix for RSBQ subscale scores**

	**GM**	**BP**	**HB**	**RP**	**BREF**	**NT**	**FA**	**WS**
General mood (GM)	1.0							
Breathing problems (BP)	.35**	1.0						
Hand behaviours (HB)	.24*	.37**	1.0					
Repetitive face movements (RP)	.28**	.39**	.29**	1.0				
Body rocking/expressionless face (BREF)	.52**	.34**	.42**	.39**	1.0			
Night-time behaviours (NT)	.68**	.34**	.24*	.43**	.41**	1.0		
Fear/anxiety (FA)	.52**	.46**	.29**	.45**	.43**	.49**	1.0	
Walking/standing (WS)	.20	−.08	.06	−.01	.11	−.00	.16	1.0

RSBQ, rett syndrome behavioural questionnaire; *P < .05, **P < .01.

### Overactivity and impulsivity

The RTT group had significantly lower total AQ scores than the contrast group as well as significantly lower Impulsivity and Overactivity subscale scores (see Table 4). Significantly lower scores were also found for the Impulsivity subscale analysed separately for immobile and mobile participants. Within the RTT sample, those with less severe clinical characteristics had higher total AQ scores (*U* = 409.0, *z* = −4.663, *P* < .001) and higher Overactivity (*U* = 608.5, *z* = −3.204, *P* < .005) and Impulsivity subscale scores (*U* = 354.5, *z* = −5.257, *P* < .001), with mean scores of respectively 19.3 (SD = 12.75), 10.5 (SD = 6.83) and 8.8 (SD = 7.45) compared to 7.7 (SD = 6.67), 6.0 (SD = 4.48) and 1.7 (SD = 3.38). There was a significant difference in Impulsivity subscale scores across broad mutation groups. Those with Early Truncating or Large Deletion mutations had a lower score (mean = 2.8, SD = 5.26) than those with Missense, Late Truncating or C-Terminal mutations (mean = 7.9, SD = 7.57) (*U* = 324.0, *z* = −3.403, *P* < .001).

**Table 4:** **Overactivity and impulsivity among the RTT and contrast groups: mean Activity Questionnaire scores (SD, range)**

	**RTT**	**Contrast**	**Mann Whitney**		
	**(** ***N*** **= 88)**	**(** ***N*** **= 66)**	***U***	***z***	***P*** **value**
Total score	14.4 (11.94, 0 to 52)	33.1 (14.92,0 to 60)	910.0	−7.14	.001
Overactivity	8.6 (6.30, 0 to 31)	18.7 (8.92, 0 to 37)	1,009.5	−6.84	.001
Impulsivity	5.8 (7.03, 0 to 24)	14.4 (7.91, 0 to 24)	1,193.0	−6.14	.001
Impulsivity (immobile)	2.8 (6.0, 0 to 24)	9.8 (7.67, 0 to 24)	262.5	−4.62	.001
Impulsivity (mobile)	8.6 (6.81, 0 to 22)	18.6 (5.48, 6 to 24)	207.0	−5.53	.001

### Mood, interest and pleasure

There were no significant differences between the RTT and contrast group on the MIPQ-S either in total or in relation to its Mood or Interest and Pleasure subscales (see Table 5). Within the RTT sample, there were also no significant total or subscale differences across clinical severity categories (severe/mild) or across mutation groups, either as originally constituted or the two broader categories. Seven children and one adult (15.6% and 2.2%, respectively) had abnormally low mood.

**Table 5:** **Mood, interest and pleasure among the RTT and contrast groups: mean MIPQ-S scores (SD, range)**

	**RTT**	**Contrast**	**Mann Whitney**		
	**(** ***N*** **= 88)**	**(** ***N*** **= 66)**	***U***	***z***	***P*** **value**
Total score	33.9 (5.76, 19 to 45)	33.7 (7.79, 4 to 47)	2,813.0	−.05	.958
Mood	19.7 (2.50, 11 to 24)	19.1 (4.19, 0 to 24)	2,753.0	−.28	.780
Interest and pleasure	14.2 (4.09, 7 to 24)	14.6 (4.57, 4 to 23)	2,676.5	−.56	.573

MIPQ-S, Mood, Interest and pleasure questionnaire short-form.

### Repetitive behaviour

There were significant differences between the RTT sample and contrast group on the RBQ in all but the Insistence on Sameness subscale (see Table 6). The contrast group had higher scores on the Stereotyped Behaviour and Compulsive Behaviour subscales and the RBQ total score. Four of the 19 items of the RBQ were not assessed as they were not relevant due to the participants’ lack of language. Moreover, on a further nine items, fewer than 10% in either group reached the clinically significant threshold. Occurrence of a clinically significant level of behaviour was greater in the contrast than RTT group in relation to five of the remaining six items: Object Stereotypies (74.2% vs. 19.3%), Body Stereotypies (62.1% vs. 30.7%), Attachment to Objects (34.9% vs. 12.5%), Repetitive Phrases (19.7% vs. 4.5%) and Preference for Routine (22.7% vs. 14.8%) Only Hand Stereotypies occurred at a clinically significant level more frequently among the RTT group (RTT = 80.7%, contrast = 71.3%).

**Table 6:** **Repetitive behaviour among the RTT and contrast groups: mean Repetitive Behaviour Questionnaire scores (SD, range)**

	**RTT**	**Contrast**	**Mann Whitney**		
	**(** ***N*** **= 88)**	**(** ***N*** **= 66)**	***U***	***z***	***P*** **value**
Total score	6.9 (4.31,0 to 24)	13.9 (7.67, 3 to 36)	1134.0	−6.01	.001
Stereotyped behaviour	5.5 (2.90, 0 to 12)	8.8 (3.22, 0 to 12)	1276.5	−5.81	.001
Compulsive behaviour	0.03 (0.31, 0 to 3)	1.5 (2.60, 0 to 12)	1876.0	−5.63	.001
Insistence of sameness	0.7 (1.64, 0 to 8)	1.2 (2.09, 0 to 8)	2474.0	−1.75	.080

Within the RTT group, there were no significant RBQ total or subscale differences across clinical severity categories or across mutation groups.

### Self-injury

Self-injurious behaviours were reported among 24 of the RTT group (28.2%, data available for *n* = 85) and 18 of the contrast group (45.0%, data available for *n* = 40). All topographies were more common in the contrast group, except for rubbing or scratching self (see Table 7). Within the RTT sample, the most common topographies were rubbing or scratching self, hitting self with part of the body and biting self. Sample participants who self-injured differed from those who did not in levels of overactivity (*U* = 451.50, *z* = −3.328, *P* < .001) and impulsivity (*U* = 484.50, *z* = −2.886, *P* < .005). Self-injury was also associated with mild clinical severity: 19 of the 24 individuals who self-injured had severity scores less than 9; 35.8% of those with mild severity were reported to self-injure compared to 16.7% of those in the severe category (*χ*^2^_(1)_ = 4.94, *P* < .05).

**Table 7:** **Percentage occurrence of self injury topographies among the RTT and contrast group who self-injure**

	**RTT**	**Contrast**	**Mann Whitney**		
	**(** ***n*** **= 24)**	**(** ***n*** **= 18)**	***U***	***z***	***P*** **value**
Hit self with body part	33.3%	55.6%	1,278.0	−3.52	.001
Hit self against surface	12.5%	38.9%	1,393.0	−3.37	.001
Hit self with object	−	50%	903.0	−6.91	.001
Bites self	33.3%	50%	1,063.0	−4.85	.001
Pull hair/skin	20.8%	44.4%	1,175.0	−4.51	.001
Rub/scratches	45.8%	33.3%	1,444.0	−2.13	.033
Insert objects	4.1%	16.7%	1,568.0	−2.36	.018

## Discussion

In this paper, we have presented behavioural data on 91 girls and women with RTT. The behavioural characteristics of all but three who had preserved language were compared to a contrast group with a range of other genetic conditions matched for gender, age, language and daily living skills. The RTT sample was drawn from a national database and was reasonably large for a study of RTT. All families whose children met the criteria were invited to participate. In particular, both children and adults were represented, the latter being the slight majority. However, the sample was skewed towards those living in the family home as an additional research purpose was to investigate the relationship between child characteristics and parental well-being. The 11 participants in out-of-family placements were, on average, older than those living in the family home (mean, 28.0 years vs. 19.5 years), albeit that the two groups were similar in age among the adults: almost all of the children lived with their parents. In addition, the two groups were similar in diagnostic distribution (82% classic vs. 75%), mean age of regression (18.5 months vs. 19.3) and mean severity score (9.0 vs. 8.5).

The response rate was low and it is not possible to assess the representativeness of the achieved sample. However, the age distribution was similar to a recent all-age, large sample (*n* = 983) study of gastrointestinal and feeding problems [25]. Moreover, over three-quarters of the sample had a positive mutation in the *MECP2* gene. Not all individuals in the sample had been tested, but in only one case diagnosed with classic RTT was a *MECP2* mutation not found. This is consistent with the literature that a mutation in the *MECP2* gene can be found in over 90% of cases with Classic RTT [3]. Consistent with other studies, the most common age of regression was between 12 and 18 months.

A further weakness of the study is that the comparison to the contrast group could only be achieved in relation to measures that had already been administered in relation to the other syndrome groups in studies conducted by the Cerebra Centre. Therefore, this study does not contain comparative data on certain RTT specific issues relevant to a potential RTT behavioural phenotype. Findings in this survey do support previous studies that hand stereotypies were almost universal and that breathing irregularities, night-time unrest, anxiety or inappropriate fear and low or changeable mood were common. However, in general, we cannot comment on their frequency relative to the contrast group.

It is possible to be more definitive about hand stereotypies as comparison of repetitive behaviour between the RTT sample and the contrast group provides further evidence for their characteristic occurrence. Hand stereotypies were the only area found to be more common among the RTT group. In other respects, stereotypic and compulsive behaviour were greater among the contrast group. However, even in relation to hand stereotypies, it should be noted that these were found among over two-thirds of the contrast group and in an equally high proportion to the RTT sample among those in the contrast group with Angelman syndrome (80%). Hence, one could conclude that hand stereotypies are characteristic of people with the degree of intellectual disability found in the two groups in this study, while it may be the particular form of hand stereotypy that is diagnostic of a particular syndrome: such as hand wringing in RTT and hand flapping in Angelman syndrome [26].

Overactivity and impulsivity were not characteristic of RTT, with scores on both AQ subscales below those found for the contrast group. Moreover, despite low or changeable mood being commonly reported in RTT, the RTT group did not differ from the contrast group with respect to either mood or interest and pleasure. A low, albeit possibly clinically important, proportion of the RTT group was found to have abnormally low mood.

Self-injury was reported only in a minority of the RTT sample and in a lower proportion than in three previous surveys (Table 1). It was also found to be less common when compared to the contrast group. The most frequent category of self-injury reported among the RTT group was rubbing or scratching self. This topography is compatible with their low level of functional hand use. Self-injury in the RTT sample was associated with overactivity and impulsivity, a finding consistent with the predictors of self-injury in certain other syndromes and in autistic spectrum disorders [16,27-29]. Overactivity and impulsivity were in turn associated with mutation group, occurring less among Early Truncating mutations and Large Deletions, which are associated with greater severity of disability. This would suggest that self-injury would occur more among individuals with severity scores in the mild range and this was found to be the case.

The ranges in the RSBQ total and subscale scores found for this RTT sample were wide and indicate that there is variability in the manifestation of the behavioural phenotype across individuals. For example, variability in Hand Behaviours and Repetitive Face Movements subscale scores were related to mutation group, with greater severity being associated with Early Truncating and Large Deletion mutations. However, the strength of positive association between the majority of subscales suggests the tendency for some individuals to have relatively high problem levels in all areas while others will generally have relatively low problem levels.

Mount *et al*. [19] and Robertson *et al*. [30] report RSBQ means for child samples. Findings for this child and adult sample differ from the Mount *et al*. survey in certain respects. Scores for the total scale and for the Breathing Abnormalities, Body Rocking and Expressionless Face, Night-time Behaviour and Fear/Anxiety subscales were similar. Subscale scores for General Mood, Repetitive Face Movements and Walking/Standing were significantly lower (respectively *t* = 3.27, 2.20, 2.33, df = 227, *P* < .01, .05, .05) while that for Hand Behaviours was significantly higher (*t* = 2.85, df = 227, *P* < .01). Where differences existed, the means given in Robertson *et al*. were closer to those reported here for all subscales other than Repetitive Face Movements.

## Conclusions

In conclusion, this study provides further evidence in support of hand stereotypies, breathing irregularities, night-time unrest and anxiety or inappropriate fear being part of a RTT behavioural phenotype, although only the former was tested against its occurrence in a contrast group and, even here, the conclusion may need to be limited to a particular form of hand stereotypy. Problems of low mood were also reported as common. However, mood and interest and pleasure were no lower than found in the contrast group. In addition, self-injury was lower than in the contrast group and was associated with factors found to predict self-injury in other groups of people with severe intellectual disabilities. Moreover, there is variability in the manifestation of problem behaviours potentially associated with the syndrome across individuals, with some more severely affected in most areas than others. Some of this variability appears to be underpinned by genetic mutation.

### Ethical approval

Ethical approval was provided by the Research Ethics Committee for Wales, Cardiff, Wales, UK.
